# Therapist-Supported Online Interventions for Children and Young People With Tic Disorders: Lessons Learned From a Randomized Controlled Trial and Considerations for Future Practice

**DOI:** 10.2196/19600

**Published:** 2020-10-23

**Authors:** Liam R Chamberlain, Charlotte L Hall, Per Andrén, E Bethan Davies, Joseph Kilgariff, Natalia Kouzoupi, Tara Murphy, Chris Hollis

**Affiliations:** 1 NIHR MindTech Medtech Co-operative, Institute of Mental Health University of Nottingham Nottingham United Kingdom; 2 Centre for Psychiatry Research Department of Clinical Neuroscience Karolinska Institutet Stockholm Sweden; 3 Stockholm Health Care Services Region Stockholm Stockholm Sweden; 4 Nottinghamshire Healthcare NHS Foundation Trust Nottingham United Kingdom; 5 UCL Great Ormond Street Institute of Child Health University College London London United Kingdom; 6 Great Ormond Street Hospital for Children NHS Foundation Trust London United Kingdom; 7 Division of Psychiatry and Applied Psychology Institute of Mental Health, University of Nottingham Innovation Park Nottingham United Kingdom; 8 NIHR Nottingham Biomedical Research Centre Nottingham United Kingdom

**Keywords:** Tourette syndrome, tic disorders, internet-based cognitive behavioral therapy (iCBT), remote therapy, therapist support

## Abstract

In recent years, research into internet-based cognitive behavioral therapy (iCBT) has suggested that therapist-guided digital interventions have greater engagement, adherence, and effectiveness than self-directed digital therapies. While research has focused on the effectiveness of, and adherence to, these interventions, less attention has been paid to their implementation in practice and what aspects of the therapist role support success. An understanding of the key factors related to the therapist role and intervention delivery is required if these iCBTs are to be applied in routine clinical care and outcomes optimized. In light of the coronavirus disease 2019 (COVID-19) pandemic, there is greater emphasis on allowing patients access to remote therapies. We report the experiences and reflections of 4 therapists and their 2 supervisors in delivering an online, therapist-supported intervention in a randomized controlled trial for children and young people with tic disorders (the Online Remote Behavioural Intervention for Tics [ORBIT] trial). Themes discussed include the importance of training, supervision, creating support documents/manuals, and record keeping. Alongside this are communication strategies used by therapists to encourage patient adherence and treatment effectiveness. These include rapport building, treatment personalization, and suggestions for overcoming non-engagement. These reflections offer important considerations for the delivery of iCBTs as well as implications associated with the implementation of these interventions in existing services and future research studies. We share thoughts on where iCBTs may sit in a stepped care model, how services may deal with comorbid conditions, and the potential role of iCBTs in collecting clinical data.

## Introduction

### Background

Tic disorders are associated with significant clinical impairment. Although behavioral therapies are an effective and acceptable treatment for these conditions, they are not always available due to a shortage of trained therapists [1].

As demonstrated in clinical trials, therapist-guided internet-based cognitive behavioral therapy (iCBT) is an efficacious format that has been successfully tested within various conditions [2]. These treatments are potentially cost-effective and can improve current service delivery by transcending barriers of time and geography. They are likely to be particularly useful when there is a clear lack of trained therapists, as is the case with tic disorders. Furthermore, it is the case that many low-to-middle-income countries have limited access to mental health services, and it is likely that iCBTs could provide cost-effective interventions that can be widely distributed [3]. Similarly, in light of the coronavirus disease 2019 (COVID-19) pandemic, there is an increasing need to offer psychological or behavioral interventions remotely to ensure continuity of care for existing patients and provide an avenue of support for the increasing mental health pressures as a result of the pandemic [4]. If therapist-supported iCBTs are to be integrated into standard clinical care, there needs to be consideration around the delivery of treatment, including the role of the therapist.

This viewpoint reports on the experiences of therapists who delivered an online, therapist-supported intervention in a randomized controlled trial for children and young people with tic disorders [5]. This paper also summarizes the lessons learned based on thoughts, reflections, and discussions between those in the therapist role and their supervisors in the trial. A more in-depth account of therapist and patient experiences is being formally evaluated [6]. We detail procedures used by the therapists within this trial that go beyond the method described in the original protocol paper [5], and suggest implications of applying iCBTs in existing services. These experiences are likely to be generalizable to other therapist-supported iCBTs, especially those set in child and adolescent mental health services and community pediatric settings.

### The ORBIT Trial

The Online Remote Behavioural Intervention for Tics (ORBIT) is a randomized controlled trial delivered in England between 2017 and 2021 [5]. In ORBIT, two 10-week, therapist-supported, internet-delivered behavioral interventions have been trialed: (1) *BIP TIC*, which is based on exposure and response prevention (ERP) principles [7]; and (2) psychoeducation on tics. In both treatments, child patients and an assigned supporter (usually a parent or caregiver) log in to an internet treatment platform and each work through 10 chapters of treatment content (Table 1). The chapters can be completed independently, but the supporter is encouraged to assist with the child’s comprehension of the material (ie, making sure they actually understand the chapters as they are being read).

**Table 1:** Chapters of the ORBIT treatments.

Chapter number	ERP^a^ treatment content		Psychoeducation treatment content	
	Young Person	Supporter	Young person	Supporter
1	Learn about tics	Introduction	Introduction	Introduction
2	More about tics	Thoughts and behaviors of supporters	Tics and Tic list	Praise
3	Practicing stopping your tics	Praise	Learning about tics	Prompts
4	Making the practice more challenging	Prompts	More than tics	More than tics
5	Continued practice	Situations and reactions	Healthy habits	Healthy habits for your child
6	School	Troubleshooting	School	School
7	Talk about your tics	Continued practice	Talking about tics with your class	Thoughts and behaviors of supporters
8	Continued practice	Continued practice	Risk and protective factors	Risk and protective factors
9	The final sprint	Continued practice	Tics and the future	Looking after yourself
10	Plan for the future	Plan for the future	Plan for the future	Plan for the future

^a^ERP: exposure and response prevention.

All patients were between the ages of 9 and 17 at the time of their baseline assessment. A formal diagnosis of tic disorder is not necessary for participation—rather they have to meet the threshold for having tics on the Yale Global Tic Severity Scale (YGTSS) [8]; further details can be found in the study protocol [5]. Differences in severity of tics is not formally considered during therapy. All patients and supporters are assigned to 1 of 3 therapists educated to at least a bachelor’s degree in a psychology-related discipline—with the highest qualification being a PhD. Typically, patients would meet their therapist once in person during their assessment appointment and would be allocated the same therapist throughout (except for absences). Contact with their therapist is asynchronous and predominantly through text-based communication within the platform (eg, messages resembling email, comments on completed worksheets). Therapists aimed to have approximately 10-20 minutes of contact per week with each dyad of child and supporter; this time was logged (by the platform) to measure the amount of support patients were needing and would help the therapists devote comparable time to each patient. This amount of contact is significantly less than might be expected in face-to-face behavioral therapy for tics (manualized as 1 hour per week for 10-12 weeks). Treatment is occasionally supplemented with telephone calls and emails outside of the platform, if the child or supporter was not accessing the treatment platform regularly—and these times were manually logged and combined with the times recorded by the platform.

The treatment content of ORBIT is delivered by the online platform (through text, illustrations, and videos), so the therapist role was twofold: (1) maximizing the adherence to and uptake of the treatment content (via problem-solving and content application); (2) offering first-line technical support. For other interventions, the role of the therapist may also involve delivering the actual intervention content, but this is not the case for the ORBIT interventions.

## Methods

### Procedure

The information reported in this paper derives from the shared experiences and insights had by the ORBIT therapist team.

The therapist team consisted of 4 ORBIT therapists and 2 supervisor therapists (TM and JK). Therapists EBD and LRC were active throughout the entirety of the treatment period of ORBIT, with therapist NK joining approximately 1 year into the treatment period following the departure of a previous therapist. Therapists EBD and LRC were assigned to supervisor JK and were based at the Nottingham site, and therapist NK (and her predecessor) was assigned to supervisor TM and was based at the London site. The supervisors would host weekly supervision sessions with their respective therapist(s), with the session minutes written electronically and distributed via email to the other therapists.

Reflections of therapist experience were collected throughout the course of the trial within these supervision records. The lead author (LRC) summarized these key reflections and this summary was approved by the remaining therapist team.

## Reflections

### Therapist Training and Supervision

Before treatment, the therapists were familiarized with the interventions and background literature on tic disorders. However, no formal or manualized training was given for treating or managing tic disorders as the ORBIT treatments were largely designed as self-help programs. Therapists were shown the basic functions of the internet platform and given access to “how-to” guides which highlighted how to complete the necessary tasks (eg, unlocking chapters). As the therapists were not required to be specially trained in therapy delivery, standard operating procedures were designed during the initial set up of the ORBIT trial to aid the therapist’s effectiveness and efficiency (Table 2).

**Table 2:** Standard therapist procedures used in the ORBIT^a^ trial.

Procedure	Perceived benefit(s)
Logging interactions between therapist and patients	Keeping track of progress and change over time. Able to manage larger caseloads. Improve therapeutic rapport.
Using a bank of standardized responses	Optimizing therapist time. Therapists responses remain aligned and treatment integrity maintained.
Recording patient feedback	Encourages reflective practice. Identifies strengths and weaknesses of current delivery practice to inform service improvement.
Patient face-to-face meeting with their therapist at baseline assessment	Rapport building and “humanizing” of therapist. An opportunity to ask questions (not treatment advice as pre-randomization) and improve perceived treatment credibility.
Including photos and audio of therapists within treatment platform	Rapport building and “humanizing” of therapist.
Using a standard protocol to prompt non-engaging patients (ORBIT messages→emails→telephone calls)	Motivates patients to engage with treatment. Stepped approach to contacting patients. Consistency in therapist contact time.

^a^ORBIT: Online Remote Behavioral Intervention for Tics (trial).

As the ORBIT treatments are delivered on standardized webpages, there is less room for therapist drift. However, as therapist attitudes and behaviors seem to influence patient outcomes [9], it is important to ensure therapists coordinate their approaches when delivering iCBTs. Author TM developed technical treatment manuals, to ensure the same standard and procedure of care be given to all patients regardless of allocated therapist. The therapists also had clinical supervision with 2 qualified and clinically experienced behavioral therapists (JK and TM). This maintained the fidelity of the treatment, while also giving direction on how to respond to patients when their queries went beyond the ORBIT interventions. For additional support, monthly peer supervision occurred between the therapists. This further aligned therapist attitudes and encouraged intertherapist consistency in content and amount of support.

Post-treatment peer and clinical supervision sessions were conducted to evaluate the impact of the therapist role and to highlight considerations for future implementation. Notable reflections within the ORBIT trial were that all patients were supported appropriately to their level of investment, indicating good overall engagement with high levels of patient motivation. Furthermore, the standard of care is believed to have been consistent throughout the trial and between patients. The therapists used these insights, alongside reflections recorded during the trial, to revise the original manuals and support documents. Revisions typically included updated motivational statements, common text communications, and ideas for troubleshooting both common and uncommon problems.

### Engaging Patients in Therapy

To promote adherence to a therapy, therapists should balance between patients feeling supported (ie, not alone) and empowering the individual to take action (ie, not passive) [10]. This can be harder to achieve in iCBT: therapists tried to balance support by giving direction without excessive pressure. In order to achieve this, patients initially devised an engagement plan, which would typically be what days they planned to log in each week. Therapists attempted to refine this week-by-week by adding detail of what they could be doing during this time. The patients’ self-report on their weekly worksheets would inform these refinements; therapists would send messages capturing significant elements for potential improvement and offering advice on how to tackle these. For example, a patient who struggled to talk about tics may be advised to attempt this task before their next date of logging in or before the next chapter would be opened. The therapist encouraged a collaborative process, asking whether they agreed with the proposed plan and requesting feedback on how they found completing the work. This was important as it seems that patients prefer iCBTs that are sensitive to their needs [10]. Furthermore, the therapists wanted to prepare the dyad to continue creating plans in the future without therapist input.

There was little discrimination in the therapists messaging patterns for the child and his/her supporter(s)—often when one was sent a message the other would be sent one shortly after. For example, if a child received a message encouraging the completion of a task, this would also be explained to the supporter with guidance on how they can support him/her with this task. The therapists thought this to be important with regard to keeping both users aware of the current plan of action, as well as maintaining the idea that their chapters were linked and should be completed collaboratively. It is notable, however, that some older children (mid-teens) had made it clear to the therapist that they wanted a degree of independence from their supporter throughout the treatment, and in these cases the collaboration was less emphasized. The content of the messages were also very similar, with slight differences being that the child would tend to receive more motivational statements (ie, praise) while the supporter would often receive more instructional messages; however, there was often a significant overlap. This same difference could also be seen between the younger and older children—older children would seem to do more work independently and therefore sought greater instructional advice, which would normally be requested by the supporter. When families (or child/supporter independently) became disengaged with the interventions, attempts to promote re-engagement were typically aimed at the supporter. Some examples of common phrases used within therapist messages include “You have done brilliantly with this, keep up the excellent work!,” “I just wanted to check in as I can see you have not logged in for a few days - how are you getting on with your chapter X task? Let me know if you need anything my end!,” “Thank you for your comments, it is very interesting to hear more about your personal experience,” and “I am sorry to hear that you have been struggling with your tics at the moment, have you spoken to your [Supporter] about this?”.

Alongside making the treatment content more applicable to patients, therapists tried to personalize their communication style. Therapists achieved this by remembering particular details, such as their hobbies or pets and using emojis that patients had used previously. Reciting these details later can reassure that the therapist is listening, which may be particularly important in iCBT, where intonation and body language are not evident. The ORBIT therapists believed that these strategies helped build a genuine therapeutic alliance in several cases.

## Future Considerations

Below we outline some of the implications of using iCBTs in routine clinical care, including where they can fit into established frameworks and how they can improve the collection of health care data, as well as outlining areas for future development.

### Implications of Applying iCBTs in Existing Services

The provision of iCBTs have a range of potential applications to improve current service delivery globally; they are a feasible way to bridge the mental health treatment gap in low-to-middle-income countries [3], as well as potentially being used as method to ensure continuity of care and delivery of mental health interventions during pandemics such as COVID-19 [4]. In standard care, they can be integrated into a stepped care model as a first-line or wait-list intervention, where they may reduce delays to accessing high-fidelity evidence-based interventions; however, this does require further evaluation. Furthermore, iCBTs offer a unique way of collecting data by containing clinically relevant outcome measures within their systems. This can improve the therapist’s ability to monitor the patient’s safety and well-being during treatment, and services can be greater informed of the needs of their users. However, to utilize the benefits of iCBT, considerations are needed on how the use of iCBTs can be incorporated into clinical training for health care practitioners.

Therapists need to be aware of the limitations of the iCBT they are delivering to ensure they stay within the boundaries of the specific treatment goals: for example, the ORBIT treatments only targeted tic conditions and do not offer targeted treatment for common co-occurring symptoms and conditions. During the trial, the therapists would advise patients to seek help from other health care professionals regarding concerns beyond the remit of the ORBIT interventions. In the future, ICBT therapists can be based within community mental health services, as this may offer opportunities for integrated care that allows for direct referrals to the relevant professionals locally. Another possibility is to operate in a more “hub and spoke”-based model: local services would refer to a central point for the specific intervention and integration back to local services for other co-occurring conditions, either during or after treatment completion, would need consideration. In translation to clinical practice settings, a broader menu of digital tools and interventions targeting a range of co-existing conditions will be needed and be accessible to the therapist and the patients from a single platform.

### Implications for Future Research

It will be important to assess which components in the therapeutic process (Table 2) mediate successful outcomes for digital interventions. A potential approach for future research would be to randomize these components using a multiphase optimization strategy [11].

A future study involving a digital platform for treatment delivery could randomize participants into slightly different versions of the same interventions where there are multiple assignment arms (intervention versions) with just 1 individual component (eg, content, duration, graphics, gamification, level of human support) differing between each. Further information related to identifying active components, which may be useful to evaluate in digital health interventions, has been published previously [12]. An important research task would be to see whether therapeutic alliance differs between therapies delivered with asynchronous support such as that offered in ORBIT and more synchronous support such as that offered in videoconferencing. Although there are existing measures of therapeutic alliance (see Himle and colleagues [13], for example), an additional research task will be to develop reliable and valid measures of the digital therapeutic alliance, its effect on outcome, and which therapist and patient factors influence this. This line of enquiry has been identified as a research priority in digital technology [14].

### Conclusion

This paper reflects on the therapist role within a randomized controlled trial currently being run in the United Kingdom with the aim of sharing guidance on the successful use of a therapist role in iCBTs. Importantly, in an attempt to promote adherence to and increase effectiveness of such therapies, this paper highlights important points for consideration when delivering remote iCBTs, including adequate therapist training, clinical supervision, flexibility, and organization. We further suggest how therapist-guided iCBTs could fit into pre-existing services. The ORBIT treatments have to date been evaluated within the context of a randomized controlled trial delivered at specialist centers. The findings of this trial are required to understand the clinical and cost-effectiveness of this approach and feasibility and acceptability among patients.

### Ethical Information and Trial Registration

This manuscript reflects on experiences from the ORBIT trial, which was approved by North West Greater Manchester Research Ethics Committee (REC reference 18/NW/0079). The ORBIT trial was prospectively registered with ISRTCN (ISRCTN70758207) and ClinicalTrials.gov (NCT03483493).

## Abbreviations

COVID-19: coronavirus disease 2019
ERP: exposure and response prevention
iCBT: internet-based cognitive behavioral therapy
ORBIT: Online Remote Behavioural Intervention for Tics
YGTSS: Yale Global Tic Severity Scale
